# Three- and Five-Year Follow-Up of a Combined Inpatient-Outpatient Treatment of Obese Children and Adolescents

**DOI:** 10.1155/2013/856743

**Published:** 2013-04-18

**Authors:** Sibylle Adam, Joachim Westenhoefer, Birgit Rudolphi, Hanna-Kathrin Kraaibeek

**Affiliations:** ^1^Faculty of Life Sciences, Hamburg University of Applied Sciences, Lohbrügger Kirchstraße 65, 21033 Hamburg, Germany; ^2^HealthBehavior.de GmbH, Lübecker Straße 52, 23611 Bad Schwartau, Germany; ^3^DAK-Gesundheit Unternehmen Leben, Produktmanagement, Vorsorge und Reha-Leistungen, Nagelsweg 27-31, 20097 Hamburg, Germany; ^4^DAK-Gesundheit Unternehmen Leben, Versorgungsmanagement-Leistungen zur Prävention, Nagelsweg 27-31, 20097 Hamburg, Germany

## Abstract

*Aim*. “The combined DAK therapy for obesity in children and adolescents” combines a 6-week inpatient with a 10.5-month outpatient treatment. The aim of the study is to evaluate whether the therapeutic achievements are maintained two and four years after intervention. *Method*. All subjects who had participated in the 12-month program in 2004/2005 were included in the follow-up study. Body weight, height, and physical fitness were assessed through direct measurements, behaviour, and quality of life by self-report questionnaires. Statistical analysis is based on an intention-to-treat analysis. *Results*. The response rate after three years was 63.4% and 42.2% after five years. Within three years, participants reduced their BMI-SDS significantly by 0.20 (SD 0.49) and by 0.15 (SD 0.51) within five years. Significant positive changes could be observed with respect to the participants eating behaviour. Similarly, the food intake, particularly the consumption of calorie-reduced beverages, increased significantly while that of nonrecommended foods decreased. Improvement was also seen in the subjective quality of life as well as several aspects of self-perception. *Conclusion*. Compared to baseline data, significant reduction of BMI-SDS and positive changes of health-related behaviours could be observed even three and five years after the start of the initial program.

## 1. Introduction

The prevalence of childhood obesity and associated comorbidities is high in developed countries and is still increasing in many countries. The German Health Interview and Examination Survey for Children and Adolescents (KIGGS) conducted by the Robert Koch Institute revealed that 8.7% of the 3- to 17-year-old children in Germany are overweight and an additional 6.3% are already obese [1]. In the United States, the prevalence of obesity and overweight has tripled in the last 30 years [2]. According to the cross-sectional analysis of Ogden and colleagues published in 2012, the prevalence of obese children and adolescents in the United States is 16.9% [3]. In some European countries, the prevalence of obesity is as high as 35% [4]. However, recently, this rise in obesity prevalence appears to be tapering off in several countries including the United States [3], Germany [5], and Greece [6].

Pediatric obesity may cause metabolic abnormalities such as hyperlipidemia, hypertension, impaired glucose tolerance, or even type 2 diabetes early in childhood [7, 8]. Recently, a review from van Vliet and coworkers compared cardiometabolic risk factors such as impaired fasting glucose/impaired glucose tolerance, high triglycerides, low HDL-cholesterol, and hypertension in overweight and obese children from different countries. In their investigation, children from Turkey, Hungary, Greece, Germany, and Poland showed the most unfavourable risk profile [9]. A study conducted in China compared overweight and obese children to normal weight children with respect to their relative risks for dyslipidemia, hypertension, diabetes mellitus, and metabolic syndrome. They estimated that obese children had a 3.3 times higher risk of developing hypertriglyceridemia, a 1.5 times higher risk for low HDL, and a 1.8 times higher risk for dyslipidemia compared to normal weight children in China [10]. 

In addition to the higher risk of developing comorbidities associated with overweight and obesity, long-term follow-up results indicate that obese children and adolescents tend to become obese adults [11]. As early as 1993, Serdula et al. reviewed 17 studies, which examined the progress of obesity from childhood to adulthood. They found that 26% to 41% of obese preschool children and 42% to 63% of school-age children became obese adults [12]. In addition, a recently published cohort study found that obese adolescents had a 16-fold higher risk of becoming severely obese adults with a BMI above 40 as compared to normal weight or overweight adolescents [13]. A 12-year retrospective cohort study from Japan revealed that compared to moderately obese children, obesity, in severely obese children, frequently progresses into adulthood, despite treatment. In addition, this study identified a gender difference; moderately obese boys had a twofold higher risk of being obese adults compared to girls [14]. 

While being obese as a child is associated with adverse health consequences as described earlier, continuing to be obese or severely obese in adulthood will lead to a cumulative effect in terms of the negative consequences on health [8], mortality [15], and quality of life [16–18]. 

Apart from the metabolic health problems, psychological and social consequences occur increasingly in overweight and obese children due to discrimination and stigmatisation because of their weight. During the last decade these problems attracted more and more public and public health interest [19]. Amongst children, teasing and bullying are the most frequent forms of stigmatization. Sources for this are not only peer groups but also family members and educators [20]. A study of high school students reported that 84% of participants observed that overweight adolescents are being teased at school, both in general and during physical activities [21]. There are indications that stigmatization may lead to psychological effects like mood and anxiety disorders, for example, depression, dysthymia, mania, generalized anxiety disorder, social phobia, and substance use disorders, like nicotine, alcohol or drug abuse, or dependence [22, 23], which in turn result in further health problems. In addition, body dissatisfaction [24] and lower self-esteem [25] are related to childhood overweight and obesity. Over and above, unhealthy eating, lower physical activity, or stress-induced pathophysiology may result from weight-related stigmatization [19].

In summary, a decrease of overweight and obesity in children and adolescents should reduce comorbidities and negative psychological and social consequences resulting in an overall improvement in the health-related quality of life and well-being. Therefore, early prevention and treatment programs are necessary [26]. 

Numerous studies have been conducted with the aim of preventing and reducing obesity in children and adolescents. Several intervention approaches have been offered, for example, internet-based programs [27] or school-based interventions [28–30], hospital-based programs [31, 32], and community-based programs [33–36], all with and without family-based intervention. 

Experts recommend that treatment programs for obese children and adolescents require a multidisciplinary approach. 

Several published studies examine the changes occurring between the beginning and the end of an intervention program [37–41], in some cases supplemented by a follow-up survey with observation periods up to one year [42–45]. However, the actual impact of obesity prevention or treatment programs would become more apparent if longer time periods could be observed.

A well-known study investigating school-based interventions in Germany is the Kiel Obesity Prevention Study (KOPS). KOPS started in 1996 as a cross-sectional and longitudinal 8-year follow-up study. Recently, results of the eight-year follow-up have been published [46]. As regards changes in the BMI-SDS, the primary outcome of the study, no significant overall intervention effect was observed. Only participants with a high socioeconomic status in the intervention group showed a significant reduction of BMI-SDS by 0.17, after 8 years.

In a controlled trial, a family-based therapeutic education program for obesity was compared to a traditional dietetic approach in 190 overweight children and their families [47]. After one year, both treatments resulted in a BMI-SDS reduction, 0.42 ± 0.8 in the dietetic group and 0.48 ± 0.6 in the therapeutic education group. After 3 years, however, only the therapeutic education program showed a significant reduction of BMI-SDS (0.44 ± 0.7), whereas dietetic intervention reached nearly baseline values again (BMI-SDS reduced by 0.01 ± 0.5). 

 In Italy, a five-year follow-up assessment of the hospital-based program MI PIACE PIACERMI combining a lifestyle centred approach, parental involvement, nutrition education, and cognitive-behavioural strategies reported significant reduction of BMI-SDS and successful behaviour change [32]. 

In 2012, an 8-year follow-up study about an outpatient program in Belgium has been published [48]. The authors draw the conclusion that a multidisciplinary cognitive-behavioural program helps the children to control their weight over a long time. 

For Germany, scarce long-term follow-up data of such intervention programs exists [49]. A study published by Reinehr and colleagues in 2007 described the successful weight reduction three years after the end of intervention of a 1-year outpatient intervention program of obese children and adolescents [50].

We previously reported the effects of the 12-month program “The combined DAK therapy for obesity in children and adolescents” by means of an uncontrolled observational study [51] and showed that the treatment effects in the intervention group differed significantly from those in the untreated (waiting-list) control group [52]. However, these reports had short-term results limited to a period of 6 and 12 months. The aim of the present study is to examine prospectively the long-term effects of this program over a period of 3 and 5 years, which corresponds to 2 and 4 years after the end of treatment, respectively. 

With the present study, we would like to address the following aims. Firstly, there is a significant lack of follow-up studies which evaluate the long-term outcome of obesity treatment programs. Secondly, the objective evidence of the sustainability of this combined inpatient-outpatient treatment program is an important contribution to the German health services. Thirdly, well-evaluated treatment programs are urgently needed for developing improved treatment regimens for overweight and obese patients to ultimately improve their well-being and health-related quality of life.

Our primary hypothesis is that the study participants will show a stabilisation or improvement of all evaluated behavioural parameters as well as the weight parameter (BMI-SDS) two and four years after the end of the intervention compared to baseline data.

## 2. Methods 

### 2.1. Treatment Program

The program “The combined DAK therapy for obesity in children and adolescents” exists since 2003 and was funded by the Deutsche Angestellten Krankenkasse (DAK), which is a large German Health Insurance Company with approximately 6,000,000 members. 

The program was designed for one year with an initial 6 weeks multidisciplinary inpatient treatment on the island of Sylt followed by a 10.5-month outpatient treatment program at the home of the obese children and adolescents. For the outpatient treatment phase, a nation-wide network of nutritionists and dieticians was established. The DAK program also included a family treatment program in which the parents were trained separately at home during the 6 weeks inpatient treatment of the children. The German guidelines for the treatment of obese children [53] and a published treatment manual [54] served as the basis for developing this program.

A more detailed description of the treatment program is published elsewhere [51, 52].

### 2.2. Participants, Study Design, and Follow-Up

**Figure 1:**
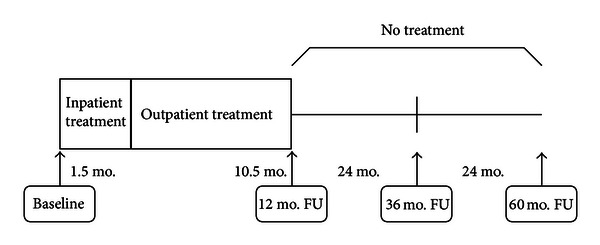
Study design (FU = follow-up; mo. = month).

Study participants were assessed (a) at the beginning of the treatment-program (baseline), (b) at the end of the 12-month combined inpatient-outpatient treatment, (c) approximately 24 months after the end of treatment, that is, 36 months after the initial start of treatment, as well as (d) 48 months after the end of treatment, that is, 60 months after the baseline measurement (see Figure 1). 

For the three- and five-year follow-up, all children and adolescents who had started with the 12-month program “The combined DAK therapy for obesity in children and adolescents” between January 2004 and June 2005 were eligible for participation. These patients were same study population as for the previously reported 12 months follow-up [51]. For inclusion in this study, candidates had to be at least 10 years old when they started the therapy program. Written informed consent by the parents or guardians of the children and adolescents was required for study participation.

In order to conduct this three- and five-year follow-up of the uncontrolled observational study, the DAK again contacted the participants' families as well as the local nutritionists and dieticians for follow-up measurement. Attempts were made to contact all 604 of the observational study participants. 

For the three-year follow-up study, 25 cases (5-year follow-up (5YFU): 43 cases) and their families were lost to follow-up despite several trials to contact them. After three years, in 51 cases (5YFU: 119 cases), it was not possible to contact the local nutritionist or dietician, who did the supervision and treatment during the 1-year program, anymore. Additionally, for the three-year follow-up, 2 local nutritionists/dieticians (5YFU: 12 nutritionists/dieticians) refused to undertake the five-year follow-up; that is, they did not to want to contact the families because of an insufficient cooperation during the treatment or because they had no time for this job. An alternative person to contact the participants or their families in all these cases could not be found.

In addition, for the three-year follow-up, 9 families could not be contacted anymore since they had moved to an unknown address, and for the five-year follow-up, this was the case with 16 families. Shifting to another city for vocational training made it impossible to contact another 12 cases for the three-year follow-up (5YFU: 2 cases).

Finally, on account of changing their Health Insurance Company—DAK, one family for the three-year follow-up and two families for the five-year follow-up could not be contacted anymore.

**Figure 2:**
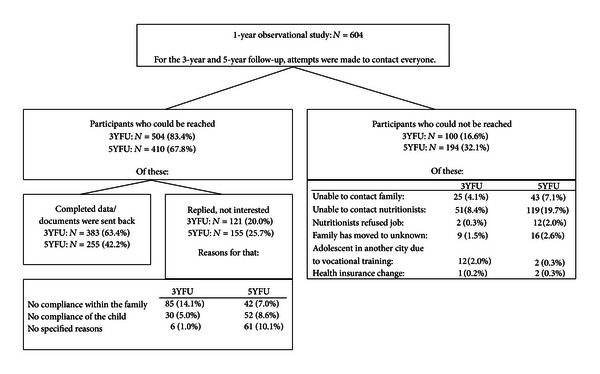
Flowchart of study participants. Calculations for the proportions (in %) are based on the originally included 604 participants. 3YFU: 3-year follow-up (36 month after baseline), 5YFU: 5-year follow-up (60 month after baseline).

As a result, data were missing in a total of 100 cases (proportion of the total 604 participants: 16.6%) for the three-year follow-up and 194 cases (proportion of the total 604 participants: 32.1%) for the five-year follow-up—all these participants could not be reached anymore (see Figure 2). 

A total of 504 children and adolescents were contacted for participation in the follow-up measurement after three years. Of these, 383 candidates attended the three-year follow-up measurement (383 of 604 total participants: follow-up rate 63.4%), while 121 candidates (20.0%) did not agree to participate any more. The most common reasons for not participating in the study were either noncompliance in the family, that is, objection against the survey by the families (14.1%), or noncompliance by the adolescents themselves (5.0%). In 6 cases, the reasons for nonparticipation were not specified (1.0%).

For the 5-year follow-up, 410 children and adolescents were contacted; of these, 255 subjects sent back the questionnaires, and 155 declared that they had no interest in participation any more (255 of 604 total participants: follow-up rate 42.2%).

As already described for the three-year follow-up, the main reasons for the participants not agreeing to participate were either objection to participate on behalf of the families (7.0%) or objection by the adolescents themselves (8.6%). Also, 10.1% of the participants did not further specify the reason for their nonparticipation at the five-year follow-up. 

After contact was established, an appointment was organised between the children or adolescents and the local nutritionists and dieticians. During this meeting, the questionnaires were filled out and weight, height, and fitness status of the candidates measured. To enhance motivation to participate, the local nutritionists and dieticians were allowed to offer a little incentive (equivalent to 10 Euros, e.g., a cinema voucher) to the families in return for participation. Data collection took place from January 2007 to June 2008 for the 3-year follow-up and from January 2009 to June 2010 for the 5-year follow-up measurement. 

### 2.3. Measurements

#### 2.3.1. Antropometric Measurements

Height and body weight were assessed directly by the dieticians. All the other parameters like behavioural characteristics, nutritional habits, physical activity or exercise status, and quality of life were assessed with the help of questionnaires. All the questionnaire items were taken from previously published manuals [54]. 

Height and weight were used to calculate BMI = kg/m^2^. BMI expressed as a standard deviation score (BMI-SDS) was calculated according to age- and sex-specific German reference data [55]. The BMI-SDS is a score which sets an individuals' BMI in context by showing how many standard deviations an individual's BMI deviates from the corresponding population mean.

Overweight was defined as a BMI above the 90th percentile, while a BMI value ≥97th percentile was considered as obese.

A weight reduction of at least 5% in one year for adults or a decrease of 0.2 BMI-SDS in children is considered as successful [58]; hence, for this study, we define a reduction of the BMI-SDS by 0.2 or more as a successful outcome.

#### 2.3.2. Eating Behaviour

 Four parameters of eating behaviour were assessed: cognitive control/restrained eating including the two subscales flexible and rigid control and disinhibition of control. We used the eating behaviour questionnaire for children which is an adapted and abridged version of the German “Three-Factor Eating Questionnaire” (TFEQ) [59]. The questionnaire includes 16 questions with 4 possible answer categories “it always applies,” “it often applies,” “it rarely applies,” and “it never applies.” 

Cognitive control/restrained eating describes the tendency to restrict food intake in order to control body weight or prevent weight gain. Flexible control is characterised by a graduated “more or less” approach to eating and weight control, which is understood as a permanent behaviour. On the contrary, rigid control is characterised by a dichotomous “all or nothing” approach to eating, where periods of strict dieting alternate with periods characterized by no weight control efforts. Disinhibition of control shows the tendency to increase food intake and overeat when exposed to emotional distress or tempting external stimuli.

Reliability for the eating behaviour questionnaire was measured by Cronbach's alpha. The scale cognitive control/restrained eating (baseline: *α* = 0.731; 3YFU: *α* = 0.735; 5YFU: *α* = 0.768), its subcomponent flexible control (baseline: *α* = 0.682; 3YFU: *α* = 0.679; 5YFU: *α* = 0.723), and also the scale disinhibition of control (baseline: *α* = 0.828; 3YFU: *α* = 0.819; 5YFU: *α* = 0.806) show good reliability at baseline and the follow-up time points. Only the scale rigid control (baseline: *α* = 0.404; 3YFU: *α* = 0.427; 5YFU: *α* = 0.426) shows a low reliability because it has only three items. 

#### 2.3.3. Food Intake/Nutritional Assessment

 To assess the children's food intake both in terms of type and quantity, a food frequency list was used. This questionnaire listed 8 food groups and the children and adolescents had to indicate how many portions of the corresponding food item or group they usually consumed using the 5 answer categories: (i) 3–5 portions per day, (ii) 1-2 portions per day, (iii) 4–6 portions per week, (iv) 1–3 portions per week, or (v) never. For the descriptive analysis, foods were grouped into recommended and nonrecommended foods and beverages according to the current German recommendations for children and adolescents [56, 57]. 

#### 2.3.4. Physical Activity and Fitness Status

 A 6-minute walking test was used to measure the participant's fitness status. The test allows for an estimation of the dynamic endurance capacity [54]. In general, a treadmill is used to test the dynamic endurance capacity, but taking into account the fact that the examined children and adolescents were spread over Germany, the test was mostly conducted on a sports field, where a defined track of 400 m was available. Since sports fields with a defined 400 m track are found throughout Germany, whereas a treadmill was not available at most places, using a sports field seems to be the best alternative to standardise the condition. The children were asked to walk briskly; they could, however, choose their own walking pace. After 6 minutes, the distance covered by the child was measured. The heart rate was also measured both before (at rest) and after the walking exercise. 

In addition, a questionnaire was used to assess the TV watching and PC habits in terms of time spent. 

#### 2.3.5. Self-Competence and Quality of Life

 The perceived self-competence of the children was measured with the help of the German questionnaire “Erfassung von Selbst- und Kompetenzeinschätzung bei Kindern (K-FSK).” This questionnaire comprises 30 items from which 5 subscales are calculated, that is, attractiveness (physical appearance), self-worth, scholastic competence, social acceptance, and self-confidence. The development of this German questionnaire by Wünsche and Schneewind [60] is based on the Perceived-Competence-Scale for Children (PCS) by Harter [61]. 

The scale attractiveness describes the degree of satisfaction with the own body and appearance; with the scale self-worth, it is asked whether the child likes her- or himself as she or he is; scholastic competence describes the children's scholastic abilities at school; the scale social acceptance describes the degree how popular a child is and how children deal with each other and the scale self-confidence assesses the confidence in the child's own behaviour. 

The items of the questionnaire are presented in a structured alternative format. For each item, two contrasting alternative statements are presented, for example, “I like going to school,” and “I do not like going to school” and for both statements the answer alternatives “really true for me” or “sort of true for me” can be chosen. 

All 5 scales of the questionnaire showed a good reliability attractiveness (Cronbach's alpha baseline 0.714; 3YFU: 0.852; 5YFU: 0.838), self-worth (Cronbachs alpha baseline 0.724; 3YFU: 0.755; 5YFU: 0.771), scholastic competence (Cronbachs alpha baseline 0.789; 3YFU: 0.820; 5YFU: 0.759), social acceptance (Cronbachs alpha baseline 0.823; 3YFU: 0.846; 5YFU: 0.847), and self-esteem in own behaviour (Cronbachs alpha baseline 0.715; 3YFU: 0.761; 5YFU: 0.773).

The quality of life was assessed with a questionnaire developed by Warschburger [62] and Warschburger et al. [63]. This questionnaire was developed for the use with overweight and obese children. This questionnaire has 11 items which describe physical-functional, emotional, and social aspects of well-being [64]. Children and adolescents are asked to answer the items related to their mood and feelings during the last two weeks. The items are answered using a 5-point Likert scale from “always” to “never” which is assigned values from 1 to 5. For analysis and interpretation, an individual mean is computed where higher values are indicative of higher quality of life [64]. This questionnaire has been validated and shows a good reliability [63]. In our study, for all time points, the questionnaire showed a good reliability (Cronbachs alpha baseline: 0.779; 3YFU: 0.819; 5YFU: 0.693). 

### 2.4. Statistical Analysis

The data was analysed using SPSS v.18.0. The statistical analysis was based on an intention-to-treat analysis and the results given as mean ± standard deviation. The missing data of the follow-up measurements were imputed using the baseline values (Return-to-Baseline or Baseline Observation Carried Forward (BOCF)). Hence, subjects lost to follow-up are considered “failures” who did not change between baseline and follow-up.

It should be noted that in some cases baseline data for certain parameters like weight, height, and fitness status (walking test) were missing. With respect to weight and height—for example—the baseline data are missing in 8 cases. For these 8 cases, a Baseline Observation Carried Forward (BOCF) analysis was not possible, and this led to a reduction of the original sample of *N* = 604 individuals to *N* = 596, when carrying out the BOCF method for analysing the data. 

We used the Wilcoxon-test for testing the statistical significance of pre-post differences in the study group and the Mann-Whitney *U*-test for comparing two independent groups. The significance level was set to *P* < 0.05, two-tailed. All other tests are considered to be exploratory analyses.

## 3. Results

The proportion of girls and boys in our study was 58.0% and 42.0%, respectively. At the beginning of the study, the average age of the children was 12.5 ± 1.35 years (range 10–15 years), and at 36 months follow-up the participants had an average age of 15.4 ± 1.31 (range 13–18 years). At the beginning of the 5-year follow-up measurement, the participants were 17.4 ± 1.35 years (range 15–20 years) old. 

### 3.1. Body-Weight Development

At the start of treatment, the average BMI was 30.4 ± 4.3 which corresponds to a BMI-SDS of 2.45 ± 0.45. In the 3 years since the beginning of the program, the BMI-SDS decreased significantly by −0.20 ± 0.49 from baseline (*z* = −9.09, *P* < 0.001). After 5 years, the BMI-SDS was reduced by 0.05 to 0.15 ± 0.51 (*z* = −6.07, *P* < 0.001) compared to baseline. The result of the 1-year evaluation for the BMI-SDS reduction was −0.35 ± 0.41 (*z* = −17.18, *P* < 0.001) (Table 1).

Although the decrease of BMI-SDS during the initial 1-year observation period was higher (−0.35 ± 0.41) compared to the 3-year (−0.20 ± 0.49) and 5-year (−0.15 ± 0.51) observation periods, 34.3% of the children and adolescents still showed successful weight reduction after 3 years, and 21.3% after 5 years, as indicated by a decrease of BMI-SDS by 0.2 or more (Table 2). 

A further analysis for assessing the BMI-SDS changes in different age groups by sex was undertaken. At the beginning of the therapy in 2004, the participants could be classified into three different age groups—(i) 10-11 years, (ii) 12-13 years, and (iii) 14-15 years. As compared to baseline measurements, the highest weight reduction after 3 and 5 years was seen in girls of the 12-13 year (at the start of the program) old age group. They reduced their BMI-SDS significantly by −0.31 ± 0.51 (*z* = −6.98, *P* < 0.001) within the 3 years and still by −0.25 ± 0.59 after 5 years (*z* = −5.13, *P* < 0.001). Girls who were 10-11 years old at the start of the therapy showed the least (nonsignificant) reduction in their BMI-SDS for the entire follow-up period of 5 years (Table 3). 

### 3.2. Eating Behaviour

Cognitive control/restrained eating and flexible and rigid control of eating behaviour all decreased slightly during the follow-up observation period after the end of the program. Yet overall, as measured from baseline, a significant improvement could still be observed for all four scales for both the 3-year follow-up and 5-year follow-up. Disinhibition and rigid control decreased significantly during the entire 3- and 5-year observation periods, and cognitive control and flexible control of eating behaviour increased significantly (Table 4). 

### 3.3. Food Intake

A division of food items into “recommend foods” and “non-recommended foods” according to the recommendations of the Research Institute of Child Nutrition (FKE) Dortmund [56, 57] shows that the intake of several nonrecommended foods and beverages was significantly reduced from baseline to 3-year and 5-year follow-up (Table 5). 

In contrast, the consumption of helpings of calorie-free and recommended beverages like (mineral) water and fruit or herbal teas increased significantly within the 3 years and also over the entire observation period of 5 years (Table 5). For other types of recommended food like whole grain products, cereals, rice and noodles, boiled potatoes, fresh fruits and vegetables, salad, and some dairy products, no significant changes could be observed. 

In addition, the consumption of meat and sausages decreased significantly from the beginning of the therapy until the 3-year and further on until the 5-year follow-up (Table 5), although the amount consumed currently is still too high. The consumption of eggs was maintained at the level of the FKE recommendations during the 3 and 5 years observation periods. 

### 3.4. Physical Activity and Fitness Status

The descriptive analysis shows no improvements for the 6-minutes walking test; the walking distance did not change significantly between baseline and both follow-ups. However, the heart rates are similar to the heart rates at the end of the therapy, one year after baseline, indicating a better physical fitness (Table 6). 

No significant differences to baseline were observed concerning self-reported activities during leisure time, time spent watching TV or using the PC. A significant reduction of TV or PC time was only seen by the end of the 1-year treatment (during the week (in hours): −0.24 ± 2.05 (*z* = −2.99, *P* < 0.010), and at the weekend (in hours): −0.23 ± 2.54 (*z* = −2.38, *P* < 0.050)). 

The question on how often the participants indulged in physical activity for at least 20 minutes per week showed a decrease during whole follow-up observation period after the end of the therapy, but compared to the baseline data, an increase could still be observed (baseline 35.6%; after 1 year/end of therapy 49.1%, 3-year follow-up 43.4%, 5-year follow-up 41.3%). 

### 3.5. Self-Perception and Quality of Life

All 5 assessed subscales of self-perception (attractiveness, self-confidence, competence at school, elf-esteem, and social acceptance) improved significantly within the 3 and 5 years. The highest changes could be measured during the 1-year program (therapy phase), while self-perception stabilised or only slightly improved after the end of the therapy phase. 

In addition, perceived quality of life improved significantly within the 3-year and 5-year observation periods. The highest increase of quality of life could be measured during the inpatient phase of the therapy. Afterwards these positive changes were maintained in most cases. Therefore, a higher quality of life after 3 and 5 years compared to baseline levels could be documented. 

At all measured time points, participants who reduced their weight successfully by 0.2 BMI-SDS units or more showed a significantly higher quality of life (Table 7). 

## 4. Discussion

The present study reported 3- and 5-year follow-up results from an observational study of an initial one-year combined inpatient-outpatient treatment of obese children and adolescents. Given the fact that obesity treatment has to focus on long-term weight reduction and maintenance rather than short-term weight loss, and given the limited number of studies reporting long-term results of obesity treatment, the present study addresses an important gap in current knowledge.

The strengths of the present study include the long follow-up duration of up to five years and a moderately large sample of 604 consecutive participants of a multidisciplinary treatment programme over 12 months.

However, before highlighting and discussing the main findings of the present study, a number of important limitations need to be pointed out. First of all, this follow-up study reports about the long-term effects of an uncontrolled study. Due to legal concerns regarding the indiscriminate access to standard treatment offered by a health insurance company, we were not able to establish a control group for the long observation period of 5 or even 3 years. However, in a previous report, we have ascertained that the short-term effects of this treatment approach over 6 months were significantly better in terms of development of body weight, health behaviour, and quality of life in the treated group as compared to untreated waiting list control group [52].

Secondly, as in other long-term follow-up studies of obesity treatment, we have a considerable portion of subjects who were lost to follow-up. Total dropout rate was 36.6% after 3 years and 57.8% after 5 years. The reasons for nonparticipation in the follow-up assessments indicate that attrition is at least partially related to unsuccessful weight and behaviour change. Nevertheless, the follow-up rates in our study may be considered as somewhat satisfying compared to other studies in the field. In an observational follow-up study of different treatment programmes in Germany, loss to follow-up was already 58.6% one year after the end of treatment and 72.5% after initial inpatient treatment [65]. In an attempt to compensate for our nevertheless large and presumably biased loss to follow-up, we consequently used an intention to treat analysis with baseline values imputed for missing values at follow-up which eventually handles dropouts as a “failure.” Thus, the estimates of treatment effects are considered conservative in the sense that true effects might be slightly better than our estimates.

In addition, several evaluated parameters were self-reports and therefore dependent on the self-perception and honesty of the participants and, hence, liable to bias. This particularly concerns the parameters eating behaviour, food intake, perceived self-competence, and quality of life. These limitations were, however, compensated for by an objective measurement of the quantitative parameter body weight, which reflects the actual physical state. 

Moreover, quite a number of behavioural and psychosocial variables were subjected to an exploratory analysis without adjusting significance levels for multiple testing. While this probably increases the number of significant results for our study, these additional results only have an informative role, that is, to explore trends in associated behavioural and psychosocial changes. The primary parameters of interest in our study are the BMI-SDS and the description of the initiating and accompanying health-related behaviour.

Taking these limitations into account, a major finding of our study was that 3 years after the start of treatment, 34.3% of the children and adolescents documented a successful weight reduction of more than 0.2 BMI-SDS units, and five years after baseline, still 21.3% of participants show a successful reduction. It should be noted that these proportions are based on all 604 patients that originally were included in the study (intention to treat principle) and that dropouts are included with an assigned BMI-SDS reduction of 0. On average, there was a significant mean reduction of BMI-SDS by −0.20 ± 0.49 after 3 years and −0.15 ± 0.51 after 5 years. This is probably less than most patients, their families, and their therapists would hope and certainly leaves room for further improvement. Nevertheless, a reduction of BMI-SDS by 0.2 has been suggested as a criterion for successful weight reduction in children and adolescents [58]. Thus, on an average, the patients maintain a successful weight reduction after 3 years even when a conservative intention to treat estimate is used as in the present study. 

Five years after baseline, the weight reduction is further attenuated to 0.15 ± 0.51 BMI-SDS units. Nevertheless, it has been shown that a weight reduction of this magnitude is already associated with a decrease of hypertension [66]. Hypertension in childhood may contribute to a higher risk for cardiovascular diseases in adulthood [67], and therefore a reduction of hypertension as a result of weight reduction during a obesity treatment program is an important effect.

Most studies focusing on long-term outcomes of childhood obesity treatment did not publish their results in the form of BMI-SDS change, and rather they indicated changes in body weight in kg or BMI. However, some studies have also published BMI-SDS data. Reinehr and colleagues (2003) [68] in a study in Germany compared the weight reduction of overweight children after one and two years, among three groups, that is, after the one-year outpatient training “Obeldicks,” after a single consultation session and in the group without treatment. After two years (the nearest to our 3-year-follow-up), the children who were trained in the outpatient treatment group showed significant reduction in BMI-SDS by −0.30 (−2.10 up to +0.46). 

Another study from Italy [47] described an even greater decrease of BMI-SDS by −0.44 ± 0.7 within 3 years, for children who had participated in a therapeutic education program. Yet the study only included overweight (not obese) children without any evident psychological problems. In addition, from that article, it was not clear whether the authors had used intention-to-treat analyses; it seems as if they reported a completer analysis, which of course yields more optimistic results.

In our study, successful weight reduction clearly showed age- and gender-specific differences. Generally, successful long-term weight reduction was considerably more frequent among girls than boys, and girls aged 12 years and older benefited more from the treatment program than younger girls. One reason for the lower success among younger patients could lie in the focus of improving self-control, which was an essential element of the therapy. Probably, the control of and responsibility for eating and exercise behaviour is more with the parents than with the children themselves. Thus, targeting the parents in the treatment for younger patients could be a useful way to improve treatment effects. Moreover, the gender differences found in the older children and adolescents suggest that more research is needed to understand how particularly boys can be supported in the change of eating and exercise behaviour.

In addition to weight reduction, a number of other significant improvements were observed. Concerning food intake, particularly the consumption of calorie-reduced beverages increased significantly and that of non-recommended foods decreased. Improvements were also seen in several aspects of perceived self-competence. However, positive changes in physical activity could not be maintained after the end of the therapy phase. 

Furthermore, our study showed that successful weight reduction is associated with improvements of quality of life. Although the group of participants who reduced their weight successfully is decreasing over the observation period, after five years, those patients with successful weight reduction maintained a better quality of life than those who did not reduce their weight successfully. 

Lower health-related quality of life has been associated with obesity in preschoolers already [16]. Obesity has been shown to be a cause for teasing and bullying in children, and these in turn can contribute to the development of psychological consequences like a depression [23]. Depressive symptoms themselves contribute to lower quality of life [69], just as obesity itself too [16]. Therefore, improvement of health-related quality of life is an important result beyond the mere weight reduction and associated health benefits. 

In Germany, recently doubts were expressed about the effectiveness of childhood obesity treatment programs, let alone the associated cost-effectiveness, given the high costs of many treatment approaches, particularly of long-term treatment. The present study shows that a life-style oriented, multidisciplinary treatment approach can achieve considerable long-term weight reduction and change of relevant health behaviours, although both far from optimum. The effects of obesity treatment on improved quality of life in addition to direct health effects related to weight reduction are worthwhile to support children and adolescents in weight reduction.

Another important aspect of the present study is related to the combination of inpatient and outpatient treatment of obese children and adolescents. Due to the structure of the German Health Care System, currently two distinct types of treatments are offered to obese patients and their families. One option being a short-term inpatient treatment of less than 3 months duration, usually the duration is 6 weeks and the treatment takes place in a specialised hospital. Often, these hospitals are located far away from the residence of the patients. The other available option is an outpatient treatment programme, conducted near the home of the patients. The duration of such programmes are considerably longer, often one year, usually with weekly therapy sessions. Results from a national observation study showed that the intensive inpatient treatment results achieve better short-term weight reduction and behaviour change at the end of treatment when compared to long-term outpatient treatment programs [65, 66]. However, long-term results one year after the end of treatment are less clear, because follow-up assessments of these inpatient treatments resulted in an enormous lost-to-follow-up rate [65]. Thus, the verified long-term rate of successful weight reduction has been considerably lower in these inpatient treatments than outpatient treatments. The average rate of verified successful weight reduction (BMI-SDS reduction more than 0.2) one year after the end of outpatient treatments appeared to be approximately 20% (intention to treat). In the present study, two years after the end of treatment, such successful weight reduction could be documented for 34% of all initial patients, and successful weight reduction was maintained over 4 years after the end of treatment for more than 20%. Thus, the combination of an initial intensive inpatient treatment followed by long-term outpatient treatments seems to combine the advantages of both approaches: substantial behaviour change during initial intensive therapy and better maintenance of changes during the transfer of behaviour changes in the everyday environment [51].

Nevertheless, the sustainability of successful weight reduction could be still better. The extended support after the end of the treatment program could be one approach to achieve better sustainability. The internet with emails, virtual meetings in chat rooms, or interactive blogs could be a promising vehicle, especially since children and adolescents nowadays feel comfortable using the internet with all its possibilities. A recent systematic review concluded that interactive computer-based interventions are effective in supporting weight loss and weight maintenance [70].

Another aspect that should be considered is the amount of exercise and intensity of family involvement in obesity treatment. A current review and meta-analysis showed that exercise and family involvement are important components of effective treatment programs [71]. Family involvement improves the probability for children and adolescents to be successful in a weight reduction program. An interesting approach to enhance family involvement in physical activity was reported in a 6-month community-based program which showed remarkable postintervention effects [72]. Children and parents received eighteen 2-hour education and exercise sessions, twice a week. This was followed by a 12-week free swimming pass for the family. Such posttreatment measure that motivates to more exercise—maybe combined with a monetary incentives—might be sensible and could enhance the sustainability of the effects of weight reduction programs. 

Regarding treatment in our study, these aspects should be considered for further improvement. Family involvement is already part of the treatment approach but could probably be intensified. In addition, anecdotal reports from staff of the initial inpatient treatment pointed out that the children were often highly motivated to continue exercise. However, back home, it was apparently difficult to maintain this motivation and continue with higher levels of exercise. Thus, generating more opportunities for exercise and supporting the motivation to use these opportunities should be addressed for further enhancement. 

## 5. Conclusion

In conclusion, our results indicate that an obesity treatment program which combines an intensive inpatient treatment with an outpatient therapy conducted in the home setting of the patient shows promising and sustainable results, not only directly after completion of the therapy phase but also 2 and 4 years after therapy.

However, in the 2 to 4 years following the active 12 months treatment program, some of the achieved changes in body weight and health behaviour were attenuated. This indicates that either longer or more effective interventions are needed to maintain the improved health behaviour trends over a longer time and consequentially to achieve a better long-term quality of life and well-being for the children and adolescents. 

## Figures and Tables

**Table 1:** Mean differences in BMI and BMI-SDS from baseline to the end of the 1-year program and to 3- and 5-year follow-up (mean (SD); *N* = 596; baseline observation carried forward).

Time period	Average change of BMI (kg/m^2^): mean (SD)	Test statistics^1^	Average change of BMI-SDS: mean (SD)	Test statistics^1^
1-year program (Baseline to end of program)	−1.75 (2.48)	Mdn = −1.38 *z* = −14.67, *P* < 0.001	−0.35 (0.41)	Mdn = −0.28 *z* = −17.18, *P* < 0.001
12th to 36th month follow-up (End of the program to 3 years after baseline)	1.90 (2.94)	Mdn = 1.63 *z* = −13.80, *P* < 0.001	0.15 (0.43)	Mdn = 0.12 *z* = −8.76, *P* < 0.001
36th to 60th month follow-up (3 years after baseline to 5 years after baseline)	0.35 (3.07)	Mdn = 0.00 *z* = −3.41, *P* < 0.010	0.05 (0.46)	Mdn = 0.00 *z* = −2.29, *P* < 0.050
Baseline to 36th month follow-up (Baseline to 3 years after baseline)	0.16 (3.22)	Mdn = 0.00 *z* = −0.88, n.s.	−0.20 (0.49)	Mdn = 0.00 *z* = −9.09, *P* < 0.001
Baseline to 60th month follow-up (Baseline to 5 years after baseline)	0.51 (3.11)	Mdn = 0.00 *z* = −3.51, *P* < 0.001	−0.15 (0.51)	Mdn = 0.00 *z* = −6.74, *P* < 0.001

^1^Test statistics calculated by Wilcoxon-test.

SD: standard deviation.

Mdn: median.

**Table 2:** Proportion (in %) of unsuccessful and successful weight reduction in the sample (*N* = 596).

BMI-SDS reduction	Baseline to end of program (1 year)	Baseline to 36-month follow-up (3YFU)	Baseline to 60-month follow-up (5YFU)
No success	31.9	56.4	74.0
Lost to follow-up	22.5	38.3	61.9
No weight loss	9.4	18.1	12.1
<0.2 BMI-SDS reduced weight	10.4	9.4	4.7
0.2–0.5 BMI-SDS reduced weight	27.2	11.6	4.9
>0.5 BMI-SDS reduced weight	30.5	22.7	16.4
Sum (total)	100.0	100.0	100.0

3YFU: 3-year follow-up (36 month/3 years after baseline).

5YFU: 5-year follow-up (60 month/5 years after baseline).

**Table 3:** Average changes of BMI-SDS by age and sex.

Average changes during…	10-11 years					
	Girls (*N* = 77)			Boys (*N* = 65)		
	Mean difference of BMI-SDS: mean (SD)	Test statistics^1^	Successful weight reduction^2^ in 10-11-year-old girls (in %)	Mean difference of BMI-SDS: mean (SD)	Test statistics^1^	Successful weight reduction^2^ in 10-11-year-old boys (in %)
1-year program (Baseline to end of program)	−0.27 (0.41)	Mdn = −0.26 *z* = −5.06, *P* < 0.001	54.4%	−0.28 (0.32)	Mdn = −0.26 *z* = −5.59, *P* < 0.001	53.8%
Baseline to 36-month follow-up (3YFU)	−0.08 (0.49)	Mdn = 0 *z* = −1.30, n.s.	27.8%	−0.11 (0.33)	Mdn = 0 *z* = −2.33, *P* < 0.050	23.1%
Baseline to 60-month follow-up (5YFU)	−0.06 (0.52)	Mdn = 0 *z* = −0.68, n.s.	17.7%	−0.03 (0.34)	Mdn = 0 *z* = −0.40, n.s.	12.3%

Average changes during…	12-13 years					
	Girls (*N* = 160)			Boys (*N* = 135)		
	Mean difference of BMI-SDS: mean (SD)	Test statistics^1^	Successful weight reduction^2^ in 12-13-year-old girls (in %)	Mean difference of BMI-SDS: mean (SD)	Test statistics^1^	Successful weight reduction^2^ in 12-13-year-old boys (in %)

1-year program (Baseline to end of program)	−0.41 (0.44)	Mdn = −0.33 *z* = −9.19, *P* < 0.001	57.2%	−0.35 (0.39)	Mdn = −0.29 *z* = −8.41, *P* < 0.001	59.2%
Baseline to 36-month follow-up (3YFU)	−0.31 (0.51)	Mdn = −0.04 *z* = −6.98, *P* < 0.001	41.0%	−−0.16 (0.48)	Mdn = 0 *z* = −3.16, *P* < 0.010	31.9%
Baseline to 60-month follow-up (5YFU)	−0.25 (0.59)	Mdn = 0 *z* = −5.13, *P* < 0.001	25.5%	−0.09 (0.51)	Mdn = 0 *z* = −1.58, n.s.	17.7%

Average changes during…	14-15 years					
	Girls (*N* = 95)			Boys (*N* = 66)		
	Mean difference of BMI-SDS: mean (SD)	Test statistics^1^	Successful weight reduction^2^ in 14-15-year-old girls (in %)	Mean difference of BMI-SDS: mean (SD)	Test statistics^1^	Successful weight reduction^2^ in 14-15-year-old boys (in %)

1-year program (Baseline to end of program)	−0.36 (0.42)	Mdn = −0.27 *z* = −7.01, *P* < 0.001	55.2%	−0.38 (0.42)	Mdn = −0.29 *z* = −6.00, *P* < 0.001	60.6%
Baseline to 36-month follow-up (3YFU)	−0.28 (0.53)	Mdn = 0 *z* = −4.70, *P* < 0.001	39.0%	−0.13 (0.47)	Mdn = 0 *z* = −2.06, *P* < 0.050	30.3%
Baseline to 60-month follow-up (5YFU)	−0.22 (0.41)	Mdn = 0 *z* = −4.70, *P* < 0.001	27.5%	−0.17 (0.52)	Mdn = 0 *z* = −52.72, *P* < 0.010	19.7%

^1^Test statistics calculated by Wilcoxon-test.

^2^“Successful weight reduction” means a BMI-SDS reduction > 0.2 BMI-SDS.

SD: standard deviation.

Mdn: median.

3YFU: 3-year follow-up (36 month/3 years after baseline).

5YFU: 5-year follow-up (60 month/5 years after baseline).

**Table 4:** Average changes in scores of eating behaviour within 3 and 5 years after baseline (mean difference (SD); *N* = 604; baseline observation carried forward).

	Disinhibition	Cognitive control	Flexible control	Rigid control
1-year program (Baseline to end of program) test statistics^1^	−0.83 (2.07) Mdn: 0 *z* = −9.29, *P* < 0.001	0.55 (2.04) Mdn: 0 *z* = −6.42, *P* < 0.001	0.59 (1.55) Mdn: 0 *z* = −8.62, *P* < 0.001	−0.04 (0.88) Mdn: 0 *z* = −1.10, n.s.
Baseline to 36-month follow-up (3YFU) test statistics^1^	−0.69 (2.01) Mdn: 0 *z* = −8.10, *P* < 0.001	0.17 (1.83) Mdn: 0 *z* = −2.22, *P* < 0.050	0.30 (1.40) Mdn: 0 *z* = −5.10, *P* < 0.001	−0.12 (0.81) Mdn: 0 *z* = −3.88, *P* < 0.001
Baseline to 60-month follow-up (5YFU) test statistics^1^	−0.53 (1.55) Mdn: 0 *z* = −7.89, *P* < 0.001	0.15 (1.51) Mdn: 0 *z* = −2.61, *P* < 0.001	0.26 (1.15) Mdn: 0 *z* = −5.34, *P* < 0.001	−0.11 (0.66) Mdn: 0 *z* = −3.95, *P* < 0.001

^1^Test statistics calculated by Wilcoxon-test.

Mdn: median.

3YFU: 3-year follow-up (36 month/3 years after baseline).

5YFU: 5-year follow-up (60 month/5 years after baseline).

Disinhibition: scores range from 0 to 8; higher scores indicate higher disinhibition.

Cognitive control: scores range from 0 to 8; higher scores indicate higher cognitive control.

Flexible control: scores range from 0 to 5; higher scores indicate higher flexible control.

Rigid control: scores range from 0 to 3; higher scores indicate higher rigid control.

**Table 5:** Changes in average number of portions or servings consumed per week (mean (SD)) from baseline to 3- and 5-year follow-up. Foods and beverages are grouped into recommended and non-recommended food and beverages based on the recommendations of the Research Institute of Child Nutrition (FKE) Dortmund [56, 57], (*N* = 604; baseline observation carried forward).

	Baseline to 36-month follow-up (3YFU)— mean (SD)	Test statistics^1^	Baseline to 60-month follow-up (5YFU)— mean (SD)	Test statistics^1^
Nonrecommended food				

White bread	−1.16 (7.27)	*z* = −4.11, *P* < 0.001	−0.96 (6.43)	*z* = −3.72, *P* < 0.001
French fries, fried potatoes	−0.40 (3.01)	*z* = −3.84, *P* < 0.001	−0.33 (2.56)	*z* = −2.70, *P* < 0.010
Vegetables in cans	−0.46 (4.45)	*z* = −2.51, *P* < 0.050	−0.30 (3.02)	*z* = −2.30, *P* < 0.050
Fruits in cans	−0.44 (3.78)	*z* = −4.01, *P* < 0.001	−0.40 (2.60)	*z* = −4.27, *P* < 0.001
Butter	−1.29 (6.10)	*z* = −5.48, *P* < 0.001	−0.82 (4.96)	*z* = −4.34, *P* < 0.001
Fruit juices	−2.42 (8.13)	*z* = −7.60, *P* < 0.001	−1.57 (6.12)	*z* = −6.39, *P* < 0.001
Crumbed fish/fish sticks	−0.45 (2.50)	*z* = −5.05, *P* < 0.001	−0.33 (1.62)	*z* = −5.12, *P* < 0.001
Snacks (not further specified)	−0.90 (3.88)	*z* = −6.07, *P* < 0.001	−0.48 (3.36)	*z* = −3.26, *P* < 0.001
Cakes and cookies	−0.93 (3.47)	*z* = −6.44, *P* < 0.001	−0.63 (3.19)	*z* = −5.43, *P* < 0.001
Chocolate and other sweets	−0.75 (4.29)	*z* = −3.60, *P* < 0.001	−0.53 (4.29)	*z* = −2.87, *P* < 0.010
Fast Food (not further specified)	−0.37 (2.92)	*z* = −2.20, *P* < 0.050	−0.16 (2.20)	*z* = −0.79, n.s.
Soft drinks	−0.64 (5.94)	*z* = −2.25, *P* < 0.050	0.00	*z* = −2.25, n.s.

Recommended food (daily intake recommended)				

(Mineral) water; tea	+1.28 (1.26)	*z* = −2.63, *P* < 0.010	+0.89 (8.97)	*z* = −2.57, *P* < 0.010

Recommended food (moderate intake recommended)				

Meat	−0.60 (5.19)	*z* = −2.91, *P* < 0.010	−0.43 (4.51)	*z* = −2.28, *P* < 0.050
Sausages	−0.45 (2.50)	*z* = −4.09, *P* < 0.001	−0.33 (1.62)	*z* = −2.75, *P* < 0.010

^1^Test statistics calculated by Wilcoxon-test.

3YFU: 3-year follow-up (36 month/3 years after baseline).

5YFU: 5-year follow-up (60 month/5 years after baseline).

Only items with significant changes are detailed (for at least one time period: baseline to 3 and/or 5 years after start of the programme). No significant changes were observed for whole grain bread, cereals, rice and noodles, boiled potatoes, fresh or deep frozen vegetables, salad, milk, cocoa, yogurt, buttermilk, quark, cheese, fish, eggs, margarine, ice, pudding, jam or honey, wine gum, coffee, and fruit juices mixed with water (1 : 2).

**Table 6:** Average changes in the walking test results after the end of the program and 3 and 5 years after start of the program (mean (SD)).

	Baseline to 12-month follow-up (end of programme)	Baseline to 36-month follow-up (3YFU)	Baseline to 60-month follow-up (5YFU)
Heart rate at rest (beats/min) test statistics^1^	−2.57 (15.75) Mdn: 0 *z* = −3.58, *P* < 0.001	−3.40 (15.35) Mdn: 0 *z* = −5.30, *P* < 0.001	−2.30 (12.05) Mdn: 0 *z* = −4.67, *P* < 0.001
Walking distance (m) test statistics^1^	6.92 (82.24) Mdn: 0 *z* = −1.60, n.s.	−3.72 (90.12) Mdn: 0 *z* = −2.00, n.s.	−3.67 (81.27) Mdn: 0 *z* = −1.60, n.s.
Heart rate (beats/min) 2 min after test test statistics^1^	−13.49 (20.53) Mdn: 0 *z* = −13.52, *P* < 0.001	−12.11 (20.02) Mdn: 0 *z* = −12.62, *P* < 0.001	−6.90 (15.44) Mdn: 0 *z* = −9.77, *P* < 0.001
Heart rate (beats/min) 10 min after test test statistics^1^	−8.87 (15.96) Mdn: 0 *z* = −12.01, *P* < 0.001	−7.80 (15.59) Mdn: 0 *z* = −10.92, *P* < 0.001	−4.54 (11.78) Mdn: 0 *z* = −8.81, *P* < 0.001

^1^Test statistics calculated by Wilcoxon-test.

Mdn: median.

3YFU: 3-year follow-up (36 month/3 years after baseline).

5YFU: 5-year follow-up (60 month/5 years after baseline).

**Table 7:** Average scores on the overall quality of life at the end of the program and after 3 and 5 years after start of the program (score range: 0–5; the higher the score the higher quality of life).

	Successful^1^			Not successful^1^			Test statistics^2^
	*N*	Mean (SD)	Median	*N*
At end of 1-year program	342	3.74 (0.70)	3.90	246	3.29 (0.72)	3.36	*U* = 26,682.50 *z* = −7.58 *P* < 0.001
36-month follow-up (3YFU)	203	3.78 (0.66)	3.91	387	3.31 (0.74)	3.36	*U* = 25,146.00 *z* = −7.19 *P* < 0.001
60-month follow-up (5YFU)	127	3.76 (0.68)	3.81	459	3.27 (0.78)	3.27	*U* = 17,860.00 *z* = −6.69 *P* < 0.001

^1^“successful” means BMI-SDS reduction ≥ 0.2; “not successful” means BMI-SDS reduction < 0.2.

^2^Test statistics calculated by Mann-Whitney *U*-test, compared group “successful” with group “not successful.”

Mdn: median.

3YFU: 3-year follow-up (36 month/3 years after baseline).

5YFU: 5-year follow-up (60 month/5 years after baseline).
